# Advantages of using a prophylactic epidural closed drain and non-watertight dura suture in a craniotomy near the “parietal site”

**DOI:** 10.1186/s41016-020-00212-2

**Published:** 2020-10-05

**Authors:** Xin Li, Jing Li, Jianfei Sui, Tuerdialimu Niyazi, Naibijiang Yalikun, Shuo Wang

**Affiliations:** 1 Department of Neurosurgery, Beijing Tiantan Hospital, Capital Medical University, No. 119 South 4th Ring West Road, Fengtai District, Beijing, 100070 China; 2 Department of Operating Room, Beijing Tiantan Hospital, Capital Medical University, No. 119 South 4th Ring West Road, Fengtai District, Beijing, 100070 China; 3 Depatment of Neurosurgery, Hetian District Hospital, No. 103 Wenhua Road, Hetian District, Hetian City, 848000 Xinjiang Uygur Autonomous Region China

**Keywords:** Craniotomy, Epidural drainage, Suction drainage, Complication, Subdural tensile hydrops, Subgaleal fluid collection, Wound infection, Intracranial infection

## Abstract

**Background:**

In neurosurgery, the necessity of having a drainage tube is controversial. Subgaleal fluid collection (SFC) often occurs, especially in a craniotomy near the “parietal site”.

This study aimed to reassess the benefit of using a prophylactic epidural drainage (ED) and non-watertight dura suture in a craniotomy near the parietal site.

**Methods:**

A retrospective review was conducted on 63 consecutive patients who underwent a craniotomy near the parietal site. The patients were divided into two groups according to different period. The deal group received ED and a non-watertight dura suture (drain group, DG), the control group that did not (non-drain group, NDG). Complications and patient recovery were evaluated and analysed.

**Results:**

Three patients (11.5%, 26) in DG and 20 patients (54.1%, 37) in NDG presented with SFC (*p* < 0.05). One patient (3.8%) in DG and three patients (8.1%) in NDG presented with subdural tensile hydrops (STH) (*p* > 0.05). Six developed an infection in NDG (four intracranial infections, one abscess, one pulmonary infection), while none in DG (*p* > 0.05) developed infection. Three (11.5%) cases in DG and one (2.7%) case in NDG had muscle strength that improved postoperatively (*p* > 0.05). Fifteen (57.7%) in DG and 14 (37.8%) in NDG had epileptic seizures less frequently postoperatively (*p* < 0.05). The average temperature (37.4 °C vs 37.6 °C, *p* > 0.05), the maximum temperature (37.9 °C vs 38.1 °C, *p* > 0.05) on 3 PODs, the postoperative hospital stay day (7.5 days vs 8.0 days, *p* > 0.05), and the postoperative medicine fee (¥29762.0 vs ¥28321.0, *p* > 0.05) were analysed.

**Conclusion:**

In patients who undergo a craniotomy near the parietal site, the prophylactic use of ED and a non-watertight dura suture helps reduce SFC, infection, and control epilepsy.

## Background

The necessity of having a drainage tube in operations is controversial. Some authors [1, 2] suggest that the blood at the operation site can be drained using a drainage tube. Wound healing is promoted, the dead cavity is eliminated. In contrast, other researchers [3–8] found that inserting a drainage tube during surgery did not significantly affect the incidence of complications at the surgical site. In neurosurgery, because the dura cannot be watertight sutured near the “parietal site”, subgaleal fluid collection (SFC) often occurs. It is difficult to compress, which affects wound healing. At the same time, subdural tensile hydrops (STH) also occasionally occur, which produce serious symptoms. However, few studies [3, 4] have suggested that drainage tubes could not assist in draining a haemorrhage or cerebrospinal fluid (CSF) effusion. We found that the drainage tubes could not be used efficaciously due to inappropriate patient choice and design in the early studies [3, 4] mentioned. Here the surgical complications of the two groups, who underwent craniotomies near the parietal site, with and without epidural drainage (ED) and non-watertight dura suture, were compared.

## Methods

### Study population

**Fig. 1:**
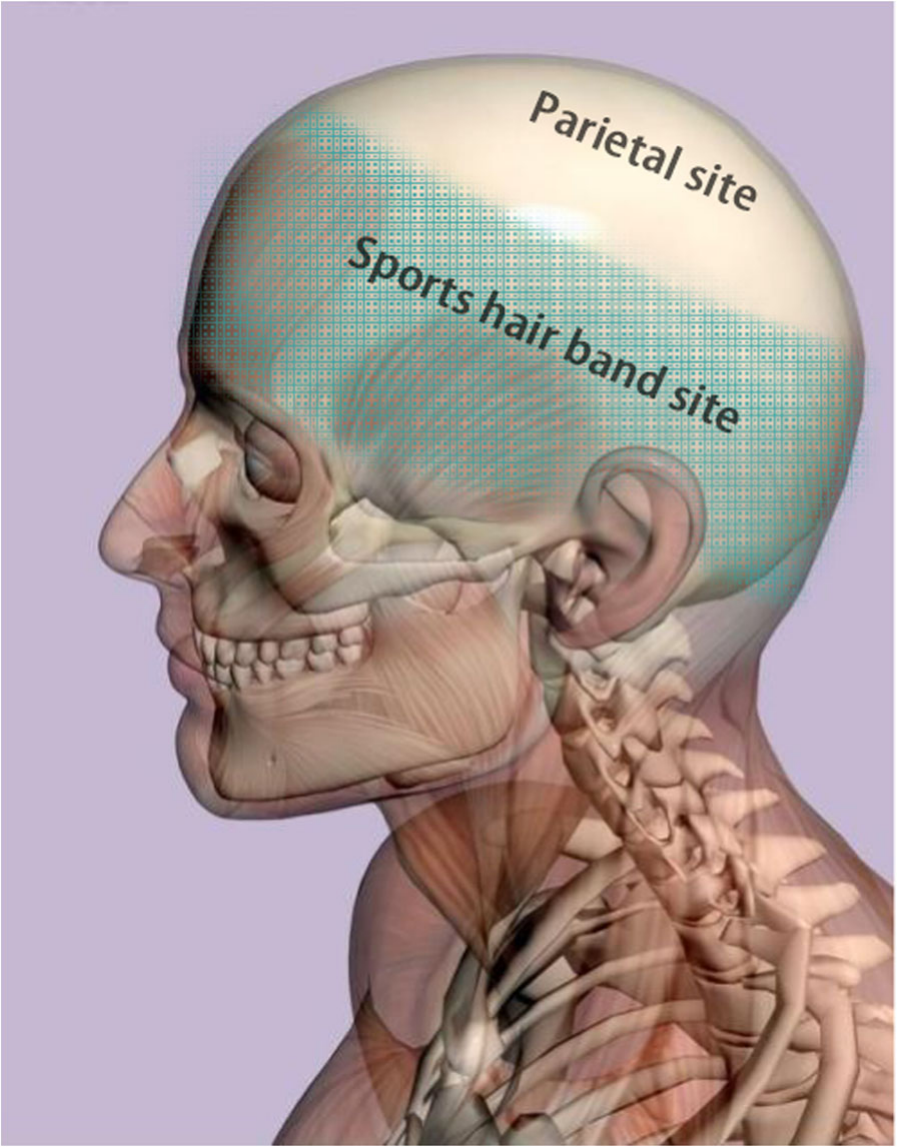
The line and the two sites. This supposed line, which is shown with a green and yellow border line, includes the bilateral temporal lines, coronal suture, and parietooccipital sulcus in the sagittal MRI. The upper part was near the parietal lobe, with little muscle attached. As the midline of the dura is not easy to watertight suture, little muscle is covered, and it is difficult to apply a compression bandage. Therefore, we named it “parietal site”, which is shown in the figure. The subdural CSF can more easily flow to the subcutaneous tissue and form SFC and not STH. The lower part is far away from the top of the head and with thick muscle attachment, which could be compression bandaged. It is similar to having a sporting hairband on your head. Therefore, we named it “Sports hair band site” in this study, which is shown in figure with green colour. The subdural fluid has difficulty flowing through the dura and muscle to the subcutaneous tissue. This means that it is easier to form STH than SFC

From March 2017 to January 2019, 63 patients with intracranial lesions underwent craniotomies near the parietal site (Fig. 1) were enrolled. The patients were divided into two groups according to different period. The deal group is performed later from February 2018 to January 2019, who received ED, and a non-watertight dura suture (DG). The control group is performed earlier from March 2017 to January 2018, who received none of them (NDG). Observation index on the recovery and complications of the two groups were gathered and analysed (Table 1).

**Table 1:** Observation indexes

Indexes	Diagnosis criteria	Significance
**Surgical site-related complications**		
**SFC**	Two doctors examined the wound flap. An obvious wave motion was present.	Main index
**STH**	CT or MRI shows a cystic effusion with an obvious occupying effect. The symptoms are high intracranial pressure, such as a headache, epilepsy, haemiplegia, aphasia, or consciousness disturbance.	A serious clinical event, may even lead to a brain hernia. Emergency decompression can be necessary if mannitol does not alleviate the symptoms.
**Infection**		
**Intracranial infection**	(1) Fever > 38.5 °C, (2) meningeal irritation sign, (3) leukocyte count in CSF > 100/ml, (4) glucose in CSF < 2.5 mmol/L.	
**Wound infection (extradural abscess)**	(1) Redness, swelling, heat, and pain in the scalp soft tissue, wound dehiscence and purulent fluid. (2) Purulent fluid was found in debridement.	
**Pulmonary infection**	(1) Symptoms of pneumonia: high fever, cough, expectoration; (2) haemogram leukocyte increased; (3) inflammation visible on chest film or lung CT.	Hemiplegia, epilepsy or consciousness disorder operatively; long-term bed rest will induce falling pneumonia.
**Epidural hematoma**	High-density space-occupying lesions on CT.	
**General condition**		
**Strength-better**	Preoperative muscle strength vs. muscle strength 1 week postoperatively, 0 unchanged or worse, 1 better.	
**Epilepsy-better**	Preoperative epileptic seizure vs epileptic seizure 1 week postoperatively, 0 unchanged or worse, 1 better.	
**T_AVG**	Average temperature from the 1st to 3rd POD.	Related to infection
**T_MAX**	Max temperature from the 1st to 3rd POD.	Related to infection
**Length of stay**	Postoperative hospital stay = total hospital stay − preoperative hospital stay.	Related to infection
**Postoperative medicine fee**	Medicine fee = total cost − high value consumables applied during operation − navigation − operation cost	Related to infection

*STH* subdural tensile hydrops, *SFC* subgaleal fluid collection, *T_AVG* average body temperature, *T_MAX* maximum body temperature

### Operation method and wound management

All patients underwent a craniotomy in the laminar airflow system operating room. The whole scalp of each patient was shaved for skin preparation. Cefuroxime (1.5 g) was used as a prophylactic antibiotic and was administered intravenously to all patients 2 h before the operation, at the induction of general anaesthesia. The surgical site was sterilised with iodine alcohol and covered with surgical film. After the tumour was removed and haemostasis was achieved, the dura mater was directly sutured by an autogenous or artificial dura. The dura suspension was performed. Apply an adhesive dressing to compress the wound lightly after finishing the closure. The ED and tightness of the dura suturing were different between the groups.

In DG, non-watertight suturing was used for the dura mater. A drainage tube (circular and transparent PVC tube, 12, Suzhou Yaxin Medical Products Co., Ltd.) was placed in the extradural space. After the craniotomy was completed, it was connected to a closed drainage bag without negative pressure suction. In NDG, the dura was sutured and repaired as tightly as possible.

### Postoperative examination and treatment

The drainage was removed within the 2nd postoperative day (POD). All patients were encouraged to start walking as soon as possible. Dehydration using mannitol Q for 8 h, rehydration, acid suppression, and antiepileptic drugs were administrated. CT was used to detect clinically silent lesions on the 1st and 7th POD. Enhanced MRI was performed on the 7th POD to evaluate the resection of the lesions. If the amount of SFC was large, it was immediately removed using a repeated aseptic puncture and syringe. A sticky dressing and light pressure bandage were applied.

### Statistical analysis

Data are reported as mean ± standard deviation (SD) or median and interquartile range (IQR). The independent samples *t* test or the Mann-Whitney *U* test, as appropriate, were used to compare patients in DG and NDG. Categorical variables were compared with the chi-square test or Fisher’s exact test. Multiple logistic regression was used to investigate the influence on in-hospital outcomes. A 2-tailed *p* value of < .05 was considered to be statistically significant. All statistical analyses were performed with SAS 9.4.

## Results

### Demographics and clinical characteristics (Table 2)

Sixty-three patients who underwent craniotomy were enrolled. The DG group included 26 (41.3%) patients, of which 8 (30.8%) were male. The mean age was 50.2 (range, 50.2 ± 16.7) years old. The pathological findings of the lesions included meningioma (14, 53.8%), cavernous haemangioma (8, 30.8%), and glioma (4, 15.4%). The average volume of the tumours was 16.9 mm^3^ (range, 6.0–40.0). The NDG group included 37 patients, of which 17 (45.9%) were male. The mean age was 49.1 (range, 49.1 ± 16.2) years old. The pathological findings of lesions included meningioma (21, 56.8%), cavernous haemangioma (12, 32.4%), and glioma (4, 10.8%). The average volume of the tumours was 24.0 mm^3^ (range, 9.0–30.0). There was no significant difference in the patients’ basic demographics and clinical characteristics that might affect complications between DG and NDG (*p* > 0.05).

**Table 2:** Baseline characteristics

Variables	Total (***N*** = 63 [100%])	No drain (***N*** = 37 [58.7%])	Drain (***N*** = 26 [41.3%])	***P*** value
**Age**	49.5 ± 16.3	49.1 ± 16.2	50.2 ± 16.7	0.7804
**Male**	25 (39.7)	17 (45.9)	8 (30.8)	0.2982
**Pathology group**				0.8658
**Meningioma**	35 (55.6)	21 (56.8)	14 (53.8)	
**CA**	20 (31.7)	12 (32.4)	8 (30.8)	
**Glioma**	8 (12.7)	4 (10.8)	4 (15.4)	
**Volume**	23.2 (9.0–32.0)	24.0 (9.0–30.0)	16.9 (6.0–40.0)	0.6727

*CA* cavernous haemangioma

### In-hospital outcomes (Table 3)

#### Surgical site-related complications

SFC was the main target of this study. Three (11.5%) patients in DG and 20 (54.1%) patients in NDG presented with SFC. There was a statistically significant difference between the two groups (*p* < 0.05). The volume of haematogenous CSF that drained was generally about 300–400 ml in DG.

**Table 3:** In-hospital outcomes

Variables	Total (***N*** = 63 [100%])	No drain (***N*** = 37 [58.7%])	Drain (***N*** = 26 [41.3%])	***P*** Value
**SFC**	23 (36.5)	20 (54.1)	3 (11.5)	0.0006
**STH**	4 (6.3)	3 (8.1)	1 (3.8)	0.6366
**Infection**	6 (9.5)	6 (16.2)	0 (0)	0.0376
**Epidural Hematoma**	2 (3.2)	2 (5.4)	0 (0)	0.5108
**Strength-better**	4 (6.3)	1 (2.7)	3 (11.5)	0.2975
**Epilepsy-better**	29 (46.0)	14 (37.8)	15 (57.7)	0.1196
**T_AVG**	37.5 ± 0.5	37.6 ± 0.6	37.4 ± 0.4	0.1332
**T_MAX**	38.0 ± 0.7	38.1 ± 0.7	37.9 ± 0.6	0.1778
**Length of stay**	8.0 (7.0–11.0)	8.0 (7.0–11.0)	7.5 (6.0–9.0)	0.2885
**Postoperative medicine fee**	29327.3 (23501.5–36592.0)	28321.0 (24290.0–36064.0)	29762.0 (23501.5–37208.0)	0.9166

*STH* subdural tensile hydrops, *SFC* subgaleal fluid collection, *T_AVG* average body temperature, *T_MAX* maximum body temperature

STH occurred less. Only one (3.8%) patient in DG and 3 (8.1%) patients in NDG presented with STH.

Two (5.4%) patients presented with a small amount of epidural haematoma, which was observed and recovered, in NDG. No patient presented with an epidural haematoma in DG.

Six (9.5%) patients got an infection. They were all in NDG and accounted for 16.7% in NDG. Four cases developed an intracranial CSF infection, one case developed a serious intracranial and extradural abscess, and debridement and osteotomy were performed; one case subsequently developed a pulmonary infection. No patients got from an infection in DG. There was a significant difference between DG and NDG after the infection outcome adjusted covariates (Table 4).

**Table 4:** In-hospital outcomes adjusted covariates

Outcome	Factor	Unadjusted analysis		Adjusted analysis	
		OR (95% CI)	***P*** Value	OR (95% CI)	***P*** Value
SFC	**Drain**				
	0	Ref.		Ref.	
	1	0.11 (0.03–0.43)	0.0016	0.05 (0.01–0.24)	0.0007
STH	**Drain**				
	0	Ref.		Ref.	
	1	0.45 (0.04–4.62)	0.5042	0.22 (0.01–2.86)	0.3172
Infection	**Drain**				
	0	Ref.		Ref.	
	1	NA		NA	NA
Epidural Hematoma	**Drain**				
	0	Ref.		Ref.	
	1	NA		NA	NA
Strength-better	**Drain**				
	0	Ref.		Ref.	
	1	4.70 (0.46–47.92)	0.1919	81467 (6.97–314E11)	0.1134
Epilepsy-better	**Drain**				
	0	Ref.		Ref.	
	1	2.24 (0.81–6.23)	0.1223	3.54 (1.07–13.26)	0.0463

*STH* subdural tensile hydrops, *SFC* subgaleal fluid collection

### General condition

Three (11.5%) cases in DG and 1 (2.7%) case in NDG had postoperative muscle strength that was better than before the operation (*p* > 0.05), named strength-better in the table.

Fifteen (57.7%) cases in DG and 14 (37.8%) cases in NDG had epileptic seizure less frequently than before the operation. There was a significant difference between DG and NDG after the epilepsy-better outcome adjusted covariates (Table 4).

The T_AVG from the 1st to 3rd POD was 37.4 ± 0.4 °C in DG and 37.6 ± 0.6 °C in NDG (*p* > 0.05). The T_MAX in the first 3 days after operation was 37.9 ± 0.6 °C in DG and 38.1 ± 0.7 °C in NDG (*p* > 0.05).

The length of postoperative hospital stays was 7.5 days (6.0–9.0 days) in DG and 8.0 days (7.0–11.0 days) in NDG (*p* > 0.05).

The postoperative medicine fee was ¥29,762.0 (23,501.5–37,208.0) in DG and ¥28,321.0 (24,290.0–36,064.0) in NDG (*p* > 0.05).

## Discussion

There is no established, objective, quantitative method and evaluation guide for surgical drainage in the literature. In neurosurgery, limited research has been conducted on using a drainage tube [3–9]. Su et al. [4] believe that the prophylactic negative pressure closed drainage under a myocutaneous flap cannot reduce the subcutaneous and epidural bleeding after a pterion craniotomy. Swollen muscles seriously affect the CT data for measuring haematomas. It is challenging to drain the blood out oozing from the muscle and fascia by drainage placed under the myocutaneous flap.

According to Zhang et al. [3], “ED cannot reduce the occurrence of epidural haematoma and subcutaneous hydrops in patients with supratentorial epilepsy”. The aim of epileptic surgery is mainly to remove the epileptic focus, which assists with internal decompression. It is different from the mechanism of meningioma and glioma. Less exudate and swelling can be observed. Therefore, epileptic surgery is also inappropriate to evaluate the efficacy of drainage.

Low intracranial pressure caused by negative pressure suction can cause a series of serious problems, including brain swelling, multiple epidural haematomas, sinus bradycardia, and cardiac arrest, all of which are mentioned in the literature [5–7, 9]. Therefore, in our study, the ED bag was placed at the level of the external auditory canal, without negative pressure suction. CSF is naturally induced by brain beat and pressure gradient. Therefore, the intracranial pressure of patients in DG is stable. No complications as mentioned above occurred in our study.

On analysing the above literature, it was clear that the designs had particular patient selection biases, or the selection of SD was not appropriate, especially regarding negative pressure suction. Therefore, our study was designed specifically for the problems of SFC, STH, and secondary infection. In our study, the site of the craniotomy was chosen as our focus. The whole cranium is supposed to be divided into two parts by a line depending on how much muscle is attached and whether it can be compression bandaged (Fig. 1). Near the parietal site, it is easier to form SFC and not STH. But near the “Sports hair band site”, it is easier to form STH than SFC.

The conditions and possible mechanisms for the formation of STH are as follows: deep and large tumour, large exudation of the cavity, the small opening of the cortex surface, no communication between the tumour cavity or ventricle, and a suture of the dura mater that is too tight. These lead to the failure of hypertonic fluid to enter into the CSF circulation through the subarachnoid space on the cortex surface or ventricle. Further, it increases the local cerebral pressure and aggravates the brain oedema. As a result, the outlet of the cavity or ventricles is narrower, and hypertonic fluid cannot flow out, leading to vicious circulation and the formation of STH. In our study, the hypertonic fluid will likely flow through the non-watertight dura and drain before the STH happens. This is preferable than removing more cortex tissue [10] for a larger CSF opening.

The results of our study have shown that the incidence of SFC in NDG (54.1%) was significantly higher than that in DG (11.5%). Further treatment, such as repeated scalp puncture, aspiration, and compressed bandage, may lead to the risk of a secondary wound infection and/or intracranial infection, or even debridement and osteotomy. It affects wound healing and brings unnecessary economic and pain burden to patients, especially in children. On the contrary, appropriate drainage could drain the haematogenous CSF. It can make the scalp and skull more attached, help the wound heal, and reduce the patients’ pain. On this basis, considering the local relative high pressure caused by exudation of the tumour cavity and swelling of the surrounding brain tissue, our study innovatively proposes the method of non-watertight dura suturing. This can make the CSF slowly transfer from the intracranial relative high-pressure site to the extradural low-pressure site by using extradural drainage through the small gap of the dura. Therefore, the occurrence of STH is low in our study.

This may be due to the craniotomy location being near the parietal site. Due to the influence of the large veins in the midline, most patients cannot achieve a watertight suture. The CSF flows out along the pressure gradient. Then, SFC occurs. In addition, repeated puncture and aspiration plays a role in the subcutaneous drainage to a certain extent. Therefore, SFC is common in NDG, and STH is rare in both groups. The craniotomy near the “Sports hair band site” was excluded from our study, making it easier to evaluate the role of the drainage tube.

**Fig. 2:**
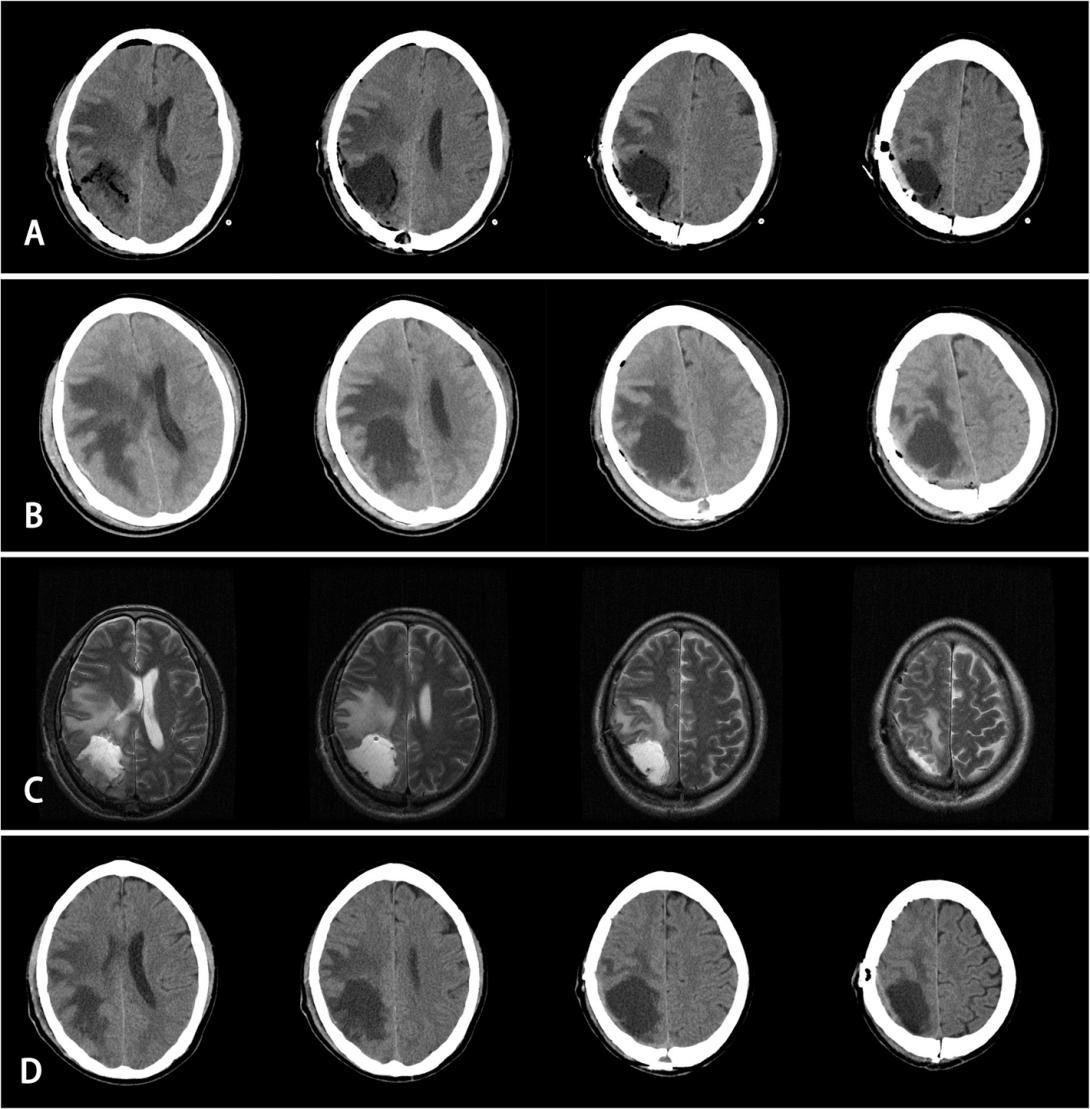
STH occurred in a patient in DG. Because of the watertight suture of the dura, only 10 ml of liquid was drawn out on the 2st POD. So the ED was pulled out. Severe headache occurred from the 3rd day. CT showed swelling of the brain tissue and STH and the mannitol q6h 250 ml was still not efficacious. After 30 ml of liquid from the subdural cavity was extracted via a puncture with a long needle through the bone hole, the symptoms were relieved. Therefore, a subsequent operation did not need to be performed. **A** CT scans showed light swelling of brain tissue. **B** CT scans showed severe swelling of brain tissue and STH on the 3rd POD. **C** MRI showed the swelling of brain tissue was reduced on the 4th POD. **D** CT scans showed light swelling of brain tissue and no headache suffered on the 10th POD

In our study, STH occurred in a special patient in DG, which proved the mechanism from the opposite side (Fig. 2). This procedure confirmed the importance of effective liquid draining. The non-watertight suture of the dura mater is as important as the drainage tube as a part of the CSF drainage system and plays a key role in reducing the occurrence of STH, which is often ignored. This precise drainage system includes the pressure gradient from the inside to the outside of dura. Relying on watertight dura suturing and bandage pressure [3], sometimes cannot effectively prevent SFC but can also lead to STH and scalp necrosis, especially in children.

The selection of ED or SD needs to be further considered. The SD was easier to lead the haemorrhage out in the cavity, avoiding the formation of a haematoma. So most articles focused on SD. Many complications were reported, especially with negative pressure suction, including pseudo hypoxic brain swelling [6], subdural haematoma along with drainage tube which was removed [8], bradycardia [5], cardiac arrest [7], and extradural haematoma [5, 11]. The cases in these articles were analysed one by one, and the reasons are summarised as follows: (1) Excessive drainage of CSF leads to low intracranial pressure. Tumour cavity drainage with negative pressure suction is widely used in general surgery, orthopaedics, and plastic surgery [12–14]. However, the intracranial cavity is different from a soft tissue cavity. It is enclosed and connected with the whole CSF circulation. It characteristically has significant fluidity. Therefore, with the outflow of CSF, the pressure will be decreased. The fluidity means that the intracranial pressure tends to average with the pressure gradient. Then, if negative pressure continues, low intracranial pressure symptoms will occur. (2) The mechanism of a subdural haematoma during extubation may be as follows: (a) rupture of a vein in the cavity. With the slow CSF extraction, the haemostatic materials such as gauze enter into the micro-hole at the head of the drainage tube. Small vessels are winded with the haemostatic materials, rupture, and haemorrhage during extubation. (a) The vein on the brain surface near the drainage tube is ruptured by friction. (b) The bleeding of the scalp artery flows into the epidural or subdural areas due to inexperience. Incomplete haemostasis of the small scalp artery during craniotomy is the reason.

Based on the above analysis, the SD is inappropriate, especially with negative pressure suction. We promote ED and a non-watertight dura suture, which is safe and effective. Of course, this is based on satisfied haemostasis of the tumour cavity. The details are as follows: (1) The dura is non-watertight sutured. (a) Dural reconstruction is necessary to avoid the low intracranial pressure caused by the over drainage of CSF and to prevent a small amount of extradural blood from flowing into the cranial. (b) Non-watertight dura suturing is important, for an unobstructed drainage system. Specifically, the gap between the sutured dura help to let the fluid flow out slowly, which ensures that the subdural hypertonic fluid flows with the pulsation of the brain. Intracavity pressure can also be regulated by the outflowing of the CSF. Therefore, it is similar to a one-way external drainage system. (2) Place ED tube. The extradural drainage tube is separated from the brain tissue by the dura. It can avoid direct contact with the tumour cavity and the vein on the brain surface, to avoid subdural haemorrhage while the tube is being removed. The ED tube can not only drain the extradural exudation but also outflow the subdual haematogenous CSF slowly to avoid STH and SFC. Of course, it is important to standardise the manipulation of placing and removing the drainage tube, to prevent iatrogenic bleeding of the scalp arterial from flowing into the epidural or subdural space.

Infection is an important concern during placing a drainage tube. Previous literature has shown that it is not caused by the haematoma, but the internalisation of skin bacteria. Long-term drainage increases morbidity and even mortality [15–18]. However, in neurosurgery, only extra ventricular drainage may cause an intracranial infection [19]. In our study, the drainage tube was located in the epidural space and removed on the 2nd POD. As long as attention is paid to aseptic operation, local infection rarely occurs. It was concluded that the incidence of intracranial, wound, and lung infection was low in DG. One abscess in the cavity and skull occurred in a case in NDG, which was resolved with debridement and external drainage. The infection rate in DG was less than in NDG, due to the following reasons: (1) It is related to the haematogenous CSF transferred to the extracranial region through a single outward drainage system, which is caused by the haematogenous exudation of the tumour cavity and the degradation of haemostatic materials. The average exudation of patients in DG was 300–400 ml. The effect of this process was similar to that of an intermittent lumbar puncture. (2) The occurrence of SFC in DG was significantly reduced, which meant that the chances of needing another scalp puncture to aspirate the fluid were also reduced, thereby reducing the risk of scalp infection and fever. (3) The decrease of haematogenous CSF stimulation, steady body temperature, the stability of the intracranial pressure, led to the reduction of postoperative symptoms, and infusion treatment. The overall state of the patients improved faster. Removing ED on the 2nd POD in DG decreased the likelihood of complications from long-term bed rest, such as lung infection and lower extremity thrombosis.

The occurrence of epidural haematoma is closely related to the thoroughness of haemostasis because haematoma solidifies quickly and is difficult to drain. Therefore, drainage has little effect, which is consistent with previously reported findings [3, 4].

The prevalence of infection, patients’ body temperature, prolongation of hospital stay, and postoperative medicine fee were studied. It was found that those in DG were all better than those in NDG. However, there was no statistical significance. Our results initially suggested that the placement of extradural drainage perhaps reduces the length of stay and expenses.

The patients selected had a craniotomy performed near the parietal site, which is close to the cerebral functioning area. We, therefore, also statistically analysed the improvement of muscle strength and the control of their epileptic seizures. Muscle strength improvement in DG was better than that in NDG, but there was no statistical difference. The control of epileptic seizures in DG was significantly better than that in NDG. The improvement of epilepsy is perhaps not only related to the decompression of the local lesion but also the reduction of haematogenous CSF, the intracranial pressure, and brain oedema.

## Conclusion

Drainage tubes are widely used in neurosurgery and can be very complex to use. Most of the literature shows that drainage tubes do not significantly affect the incidence of complications in the operation site. However, prior research did not undertake a more detailed grouping study according to specific situations. Based on previous studies, we conducted a more targeted study on the role of ED and non-watertight dura sutures in patients with craniotomy, located near the parietal site. It has a clear effect on reducing SFC, reducing infection, and controlling epilepsy. It is worth using in neurosurgery. However, its preventive effect on STH needs to be studied with a larger sample size.

## Data Availability

The datasets used and/or analysed during the current study are available from the corresponding author on reasonable request.

## Abbreviations

F: Female
M: Male
CT: Computed tomography
DG: Drain group
NDG: Non-drain group
ED: Epidural drainage
SD: Subdural drainage
POD: Postoperative day
T_AVG: Average body temperature
T_MAX: Maximum body temperature
CSF: Cerebrospinal fluid
STH: Subdural tensile hydrops
SFC: Subgaleal fluid collection
